# Identifying influential metrics in the combined metrics approach of fault prediction

**DOI:** 10.1186/2193-1801-2-627

**Published:** 2013-11-23

**Authors:** Rinkaj Goyal, Pravin Chandra, Yogesh Singh

**Affiliations:** Guru Gobind Singh Indraprastha University, Sector 16 C, Dwarka, Delhi, 78 India; University School of Information and Communication Technology, Guru Gobind Singh Indraprastha University, Sector 16 C, Dwarka, Delhi, 78 India

**Keywords:** Fault prediction, Interaction, Object oriented metrics, Regression, Stepwise regression

## Abstract

Fault prediction is a pre-eminent area of empirical software engineering which has witnessed a huge surge over the last couple of decades. In the development of a fault prediction model, combination of metrics results in better explanatory power of the model. Since the metrics used in combination are often correlated, and do not have an additive effect, the impact of a metric on another i.e. interaction should be taken into account. The effect of interaction in developing regression based fault prediction models is uncommon in software engineering; however two terms and three term interactions are analyzed in detail in social and behavioral sciences. Beyond three terms interactions are scarce, because interaction effects at such a high level are difficult to interpret. From our earlier findings (Softw Qual Prof 15(3):15-23) we statistically establish the pertinence of considering the interaction between metrics resulting in a considerable improvement in the explanatory power of the corresponding predictive model. However, in the aforesaid approach, the number of variables involved in fault prediction also shows a simultaneous increment with interaction. Furthermore, the interacting variables do not contribute equally to the prediction capability of the model.

This study contributes towards the development of an efficient predictive model involving interaction among predictive variables with a reduced set of influential terms, obtained by applying stepwise regression.

## Background

Fault prediction models based on different modelling techniques have been widely used to improve software quality for the last three decades. Out of the many modelling techniques used by researchers, regression and its variants are still drawing a major portion of the attention of research communities (Basili et al. 1996; Denaro et al. 2003; Yu 2012; Bibi et al. 2008; Thwin and Quah 2005; Briand et al. 2000; Khoshgoftaar et al. 2002; Gyimothy et al. 2005). Comparison of regression with other evolutionary algorithm based techniques has also been appraised as well (Raj Kiran and Ravi 2008; Radjenovic et al. 2013).

The application of regression analysis focuses on identifying potential complexity metrics and building relationship models that are capable of identifying faults-prone software modules.

No single set of metrics exists which can be applied to all projects equally. Therefore by taking failure [scenarios] and their correlation into account within a project, the capability to design an improved prediction model can be achieved by combining metrics ( 2006).

In the recent literature, the benefits and comparative advantages of using a combination of source code metrics to predict bugs, has been illustrated by (D’Ambros et al. 2012) and (Okutan and Yildiz 2012). However, combining metrics may lead to interactions among metrics which has not yet been properly dealt within software engineering literature, though it has been reported in other areas of the sciences and engineering.

This issue has been highlighted in our previous study (Goyal et al. 2013) in which we developed eight different models by considering two types of metrics i.e. Chidamber and Kemerer (CK) and other object oriented (OO) metrics (Chidamber and Kemerer 1994). These models describe different possibilities of two-term interaction in which the first four models take combinations of CK and OO into consideration. The four remaining models consider CK, OO and their combination separately with or without quadratic terms.

Through our earlier findings, we statistically established that the full-interaction model in which, linear two-term interaction with self-interacting terms outperforms other models.

Though the models developed in the previous study were statistically effective, the large number of predictive variables arising from interaction may lead to the over-fitting of data, thereby giving rise to prediction errors.

In this study our goal is to select the most influential metrics, derived through the interaction, since all candidate complexity metrics may not have equally resolute predictive powers. In order to reduce the dimensionality of data a feature selection technique needs to be utilized. For the purpose of this paper, we have used stepwise regression.

Through applying stepwise regression a subset of predictors that optimally models the measured responses has been computed, which yields the most influential combination of predictive variables.

## Data and mathematical methods used

The following methodology has been implemented in order to select those suitable variables, from amongst the chosen predicting variable set, taken into account in this study.

### Selection and structure of the dataset

For the purpose of validating the method and mechanism proposed in this paper we have taken a publicly available bug prediction dataset (D’Ambros et al. 2010) available at (http://bug.inf.usi.ch). Amongst other statistical data available in this dataset, we have taken into consideration 6 CK (Chidamber and Kemerer) metrics and 11 OO (Object Oriented) metrics, for five software systems i.e. Eclipse, Mylyn, Equinox, PDE and Lucene. Within the purview of this paper, however, we use single version approaches of bug prediction, assuming that the current design and behaviour of the program influences the presence of future defects, and thereby does not require the history of the system (D’Ambros et al. 2010). Table 1, below describes the metrics of the dataset used in this study.

**Table 1:** **Description of class level source metrics**

CK Metric (Chidamber and Kemerer^1994^)	Interpretation
Coupling between bbject classes (CBO)	Investigates the coupling between classes by taking the dependency of one class with other classes into consideration.
Depth of the inheritance tree (DIT)	Investigates the complexity of inheritance hierarchy by counting ancestor levels in the inheritance tree.
Lack of cohesion metric (LCOM)	Investigates cohesion with a class by measuring the dissimilarity of methods.
Response for the classes (RFC)	Investigates the coupling between classes by calculating the sum of the number of local methods and the methods that can be called remotely.
Weighted methods per class (WMC)	Investigates the complexity of class by summing up the complexity of methods.
Number of children (NOC)	Investigates complexity of inheritance hierarchy by counting the number of immediate subclasses of a class.
**OO (Object oriented)**	**Interpretation**
NOA	Number of attributes.
FanIn	Number of other classes that reference the class.
FanOut	Number of other classes referenced by the class.
NOAI	Number of attributes inherited.
NLOC	Number of lines of code.
NOM	Number of methods.
NOMI	Number of methods inherited.
NOPRA	Number of private attributes.
NOPRM	Number of private methods.
NOPA	Number of public attributes.
NOPM	Number of public methods.

### Multiple linear regression (MLR)

Models the relationship between two or more independent variables (x_1_, x_2_, …, x_k_) with the dependent variable (y) (Eq. 1), and can be expressed as Data = Fit + Residual (Pedhazur 1997; Cohen et al. 2003).1

where α_0_ = intercept term, α_1_, α_2_: coefficients for the independent variables and ϵ is a random error component.

### MLR with interaction

In MLR, *Y* is a linear function of all *k* input variables. However to bring an additional level of regression (Eq. 2), the interaction between variables ought to be considered. This in turn provides a synergistic effect of combined predictors. Like with two interacting variables x_1_ and x_2_ the model would be as follows:2

where α_0_ = intercept term, α_1_, α_2_: coefficients for the independent variables and α_12_: coefficient for the interaction term; x_1_, x_2_: values taken by the independent variables.

### Formation of a set of linearly interacting terms

- Step 1: Consider n variables i.e. x_1_ to x_n_.
- Step 2: For a variable x_1_, consider pairwise interaction with remaining n-1 variables.
- Step 3: Repeat step 2 for all other remaining variables as well.

The systematic execution of steps 1–3 result in **[n (n-1)/2] + n** number of variables arranged as a triangular matrix with the diagonal values as zero, since the self-interaction between variables resulting in quadratic terms are not being considered here. For example for 17 variables, the set would comprise of [(17 *16) /2] +17 = 153 linearly interacting terms.

### Triangular Matrix representing interacting terms



The total number of terms, including linear interaction of different kinds of metrics considered (i.e. CK, OO and their combinations) is as follows:

(i) For CK metric analysis: **21**
(ii) For OO metric analysis: **66**
(iii) For CK + OO analysis: **153**

### Experimental design and statistical measures used

In our experiment, we do cross validation with 50 fold 90%-10% splits of the training and validation sets, which further validates the values of statistical measures reported by D’Ambros et al. ( 2010) for CK and OO metrics in isolation. These have been implemented and simulated in the Matlab 7.9.0 (R2009b) environment. Table 2, below highlights the empirical aspect of the dataset provided for a single version CK-OO metrics.

**Table 2:** **Empirical aspect of the dataset**

Dataset	Release	No. of records	No. of classes	No. of attributes	No. of defects	Percentage of defect
Mylyn	3.1	1862	2196	17	340	18.26
PDE	3.4.1	1497	1562	17	341	22.78
Eclipse	3.4	997	997	17	374	37.51
Equinox	3.4	324	439	17	244	75.31
Lucene	2.4.0	691	691	17	97	14.04

To compare the performance of the models developed, we present R^2^, Adjusted R^2^ values as statistical measures. The R^2^ measures the percentage of explained variation in the dependent variable of a predictive model by taking every independent variable into consideration. Its value lies in between 0 and 1, with a value closer to one indicating the strong predictive capability of the model developed. However, value of R^2^ can be increased by including more independent variables which may not be having sufficient explanatory power. Thus, the value of R^2^ needs to be adjusted for the degree of freedom. The adjusted R^2^ is a preferred statistical measure to ascertain the fitness of the model; it quantifies the percentage of variance explained by only those independent variables which actually touch on the dependent variable ( Runkler 2012). A value of Adj. R^2^ approaching to 1 indicates better performance of predictive models.

R^2^ and Adj. R^2^ can be computed as follows (Refer Eq. 3 & 4):34

where SSE = Sum of squared error of the dependent variable

SST = Sum of squared derivation of the dependent variable

n = Sample size

p = Number of predictors (independent variables)

### Step-wise regression (SWR)

In regression analysis with a long list of independent variables, some of which may not be useful predictors, the purpose is to find the best subset of independent variables. Trying out all subsets would result in too large a number of possibilities. For example, in our experiment the number of possibilities would be 2^153^ -1, which is too a large number to compute within the scope of this model, thereby making the problem computationally intractable.

The stepwise model–building technique ( Draper and Smith 1981) could be one potential solution to this problem. Within this technique the predictor variables are included one at a time, depending upon whether the included variable increases the adjusted R^2^ or not. Initially, the R^2^ value of each variable is considered independently, following which stepwise regression is implemented, starting with that variable that has the highest value of R^2^ and moving on to the next variable with next highest R^2^ value. This process continues until the adjusted R^2^ starts decreasing. The adjusted R^2^ is used as a “stepping” criterion here.

## Results and discussion

### The repercussion of considering interaction amongst metrics in the development of a predictive model

To appropriately highlight the importance of interaction, the statistics generated from all five modules of the dataset considered, along with number of corresponding variables are shown in Table 3. CK (WOI) refers to CK metrics without interaction and CK (WI) considers CK metrics with interaction. We have used similar terminology with the other metrics considered as well. The data in Table 3 adequately reflect that after considering the interaction with CK metrics, there is a significant improvement in the adjusted R^2^ value for all software modules, while correspondingly, also resulting in an undesired increase in the number of variables i.e. from 6 to 21. Similarly for OO metrics, an improvement in adjusted R^2^ results in an undesired increase in the number of variables from 11 to 66. Taking a combination of CK and OO metrics returns an even greater value of adjusted R^2^ across all five software modules, but this improved predictive power is achieved at the cost of the variables increasing from 17 to 153.

**Table 3:** **Statistical measures with (WI) and without (WOI) interaction of metrics**

Metrics	Mylyn		PDE		Eclipse		Equinox		Lucene		No of variables
	R^2^	Adj R^2^	R^2^	Adj R^2^	R^2^	Adj R^2^	R^2^	Adj R^2^	R^2^	Adj R^2^	
**CK (WOI)**	0.1186	0.1157	0.0614	0.0576	0.3856	0.3818	0.5652	0.5570	0.3838	0.3784	6
**CK (WI)**	0.1877	0.1784	0.0976	0.0848	0.5318	0.5217	0.6826	0.6605	0.4202	0.4020	21
**OO (WOI)**	0.1757	0.1708	0.6271	0.6243	0.4129	0.4064	0.6320	0.6191	0.2457	0.2335	11
**OO (WI)**	0.3516	0.3277	0.7049	0.6912	0.6210	0.5941	0.8349	0.7925	0.5774	0.4996	66
**CK + OO (WOI)**	0.2024	0.1950	0.6439	0.6398	0.4280	0.4190	0.6906	0.6730	0.4049	0.3899	17
**CK + OO (WI)**	0.4784	0.4316	0.7683	0.7419	0.7933	0.7558	0.9384	0.8830	0.6847	0.5948	153

In Table 3, Mylyn exhibits lower values of Adj. R^2^ when compared to other software modules for CK and OO both (with and without interaction). This may be due to the fact that the procedural code complexity of the methods of a class has not been taken into account and this study focuses only on object oriented metrics.

### Obtaining a reduced set of influential terms

In order to find the best subset of interacting variables, which provides an enhanced explanatory and predictive power, stepwise regression (SWR) was performed up to 10% of the threshold of the improved Adj. R^2^. The following Tables 4, 5 and 6 (for five software modules) show a reduced number of interacting metrics. Initially, SWR was performed for the combination of CK and OO metrics up to a threshold level of 10% of corresponding adjusted R^2^ value of each software module. For Mylyn 36 metrics are sufficient to be considered out of 153 total possibilities. Similarly, for other software modules also, we observe a significant reduction in the number of metrics to be considered relevant, as is evident from Table 4.

**Table 4:** **List of reduced set of metrics for the combination of CK and OO metrics**

Total number of interacting metrics = 153		
Software module	Reduced set of metrics	No. of reduced metrics
**Mylyn**	CBO, RFC, NOA, FanIn, FanOut, CBO + DIT, CBO + RFC, CBO + NLOC, CBO + NOM, LCOM + NOA, LCOM + FanOut, LCOM + NOPRA, LCOM + NOPRM, RFC + NOM, RFC + NOPRM, WMC + NOC, WMC + NOPRM, WMC + NOPM, NOC + NOPRM, NOA + FanOut, FanIn + FanOut, FanIn + NOMI, FanIn + NOPRM, FanIn + NOPA, FanIn + NOPM, FanOut + NLOC, FanOut + NOPM, NLOC + NOPRM, NOPRA + NOPA, NOPRA + NOPM, NOPRM + NOPM, NOPA + NOPM, DIT + WMC, NOC + FanOut, FanOut + NOPRM, FanOut + NOMI	**36**
**PDE**	RFC, NOA, NOPRM, CBO + LCOM, DIT + LCOM, DIT + WMC, NOA + NOPRA, NOA + NOPA, FanIn + FanOut, FanOut + NLOC	**10**
**Lucene**	LCOM, NOPRA, LCOM + WMC, LCOM + NOPA, RFC + NOC, RFC + FanOut, RFC + NOPM, WMC + NOPM, NOC + NOMI, NOC + NOPA, FanOut + NLOC, NOMI + NOPRA, NOMI + NOPRM, NOMI + NOPM, NOPRA + NOPA, NOPRM + NOPM	**16**
**Equinox**	CBO, WMC, NOMI, NOPRA, NOPRM, CBO + DIT, DIT + FanOut, LCOM + NOC, LCOM + NOAI, RFC + FanIn, WMC + FanOut, NOC + FanOut, NOA + NOAI, FanIn + FanOut, FanIn + NOPRA, FanIn + NOPRM, FanIn + NOPA, NLOC + NOMI, NOM + NOPRM	**19**
**Eclipse**	CBO, RFC, NOPM, CBO + FanOut, DIT + WMC, DIT + NLOC, DIT + NOM, LCOM + NOAI, RFC + NOC, RFC + NOAI, RFC + NOPRA, RFC + NOPRM, RFC + NOPA, WMC + FanIn, WMC + NOAI, WMC + NLOC, WMC + NOM, WMC + NOPRM, WMC + NOPM, NOC + FanIn, NOC + FanOut, NOC + NLOC, NOA + NOPM, FanIn + NOPA, FanOut + NOAI, FanOut + NOM, FanOut + NOPM, NOAI + NLOC, NOAI + NOMI, NOAI + NOPRM, NLOC + NOPRA, NLOC + NOPRM, NLOC + NOPA, NOM + NOPRM, NOM + NOPA, NOMI + NOPA	**36**

**Table 5:** **List of the reduced set of metrics considering CK metrics only**

Total number of interacting metrics = 21		
Software module	Reduced set of metrics	No. of reduced variables
**Mylyn**	CBO, RFC, RFC + WMC, RFC + NOC, WMC + NOC	**5**
**PDE**	RFC, DIT + WMC	**2**
**Lucene**	LCOM, CBO + RFC	**2**
**Equinox**	CBO, CBO + LCOM, LCOM + NOC	**3**
**Eclipse**	CBO, RFC, CBO + RFC, DIT + LCOM, DIT + WMC, RFC + WMC, RFC + NOC	**7**

**Table 6:** **List of the reduced set of metrics considering OO metrics only**

Total number of interacting metrics = 66		
Software module	Reduced set of metrics	No. of reduced variables
**Mylyn**	NOA, FanOut, NOPRM, NOA + FanOut, FanIn + NOPA, FanOut + NOM, FanOut + NOMI, FanOut + NOPRM, FanOut + NOPM, NLOC + NOPRM, NOM + NOPRA, NOM + NOPRM, NOPRA + NOPRM, NOPRA + NOPM, NOPRM + NOPM, NOMI + NOPRA, NOMI + NOPRM	**17**
**PDE**	NOA, FanOut, NLOC, NOA + NOPRA, FanOut + NOMI, NOMI + NOPRM	**6**
**Lucene**	NOM, NOA + FanIn, FanIn + NOPRA, FanIn + NOPA, FanOut + NOM, FanOut + NOPA, NOMI + NOPRA, NOMI + NOPA	**8**
**Equinox**	FanIn, FanOut, FanIn + NLOC, FanIn + NOPRA, NLOC + NOMI	**5**
**Eclipse**	NOA, FanIn, FanOut, NOAI, NLOC, NOPA, NOA + FanIn, NOA + FanOut, NOA + NLOC, FanIn + FanOut, FanIn + NLOC, FanIn + NOPRM, FanOut + NOM, FanOut + NOPRA, FanOut + NOAI, NOAI + NLOC, NOAI + NOPA, NLOC + NOPA, NLOC + NOPM, NOMI + NOPA, NOPRA + NOPA, NOPRM + NOPM	**22**

SWR was then conducted for CK and OO metrics in isolation. The total number of possibilities is 21 in the case of CK and 66 for OO. Again, we can observe a significant reduction in the number of relevant interacting metrics as is evident from Tables 5 and 6.

### Superset of interacting terms for all software modules

The superset of a reduced number of metrics is obtained with the intent to construct a cross-project and robust fault prediction model, which adequately acts upon all five different software modules ( Peters et al. 2013). It is obtained by computing the union of the set of the reduced number of metrics, for all five software modules. The superset of interacting metrics for CK, OO and their combination is depicted in Table 7.

**Table 7:** **Superset of all metrics included in all five software modules**

Metrics	Superset of reduced and influential metrics	No. of reduced metrics
**CK (WI)**	CBO, LCOM, RFC, CBO + LCOM, CBO + RFC, DIT + LCOM, DIT + WMC, LCOM + NOC, RFC + WMC, RFC + NOC, WMC + NOC	**11**
**OO (WI)**	NOA, FanIn, FanOut, NOAI, NLOC, NOM, NOPRM, NOPA, NOA + FanIn, NOA + FanOut, NOA + NLOC, NOA + NOPRA, FanIn + FanOut, FanIn + NLOC, FanIn + NOPRA, FanIn + NOPRM, FanIn + NOPA, FanOut + NOM, FanOut + NOMI, FanOut + NOPRA, FanOut + NOPRM, FanOut + NOPA, FanOut + NOPM, NOAI + NLOC, NOAI + NOPA, NLOC + NOMI, NLOC + NOPRM, NLOC + NOPA, NLOC + NOPM, NOM + NOPRA, NOM + NOPRM, NOMI + NOPRA, NOMI + NOPRM, NOMI + NOPA, NOPRA + NOPRM, NOPRA + NOPA, NOPRA + NOPM, NOPRM + NOPM	**38**
**CK + OO (WI)**	CBO, LCOM, RFC, WMC, NOA, FanIn, FanOut, NOMI, NOPRA, NOPRM, NOPM, CBO + DIT, CBO + LCOM, CBO + RFC, CBO + FanOut, CBO + NLOC, CBO + NOM, DIT + LCOM, DIT + WMC, DIT + FanOut, DIT + NLOC, DIT + NOM, LCOM + WMC, LCOM + NOC, LCOM + NOA, LCOM + FanOut, LCOM + NOAI, LCOM + NOPRA, LCOM + NOPRM, LCOM + NOPA, RFC + NOC, RFC + FanIn, RFC + FanOut, RFC + NOAI, RFC + NOM, RFC + NOPRA, RFC + NOPRM, RFC + NOPA, RFC + NOPM, WMC + NOC, WMC + FanIn, WMC + FanOut, WMC + NOAI, WMC + NLOC, WMC + NOM, WMC + NOPRM, WMC + NOPM, NOC + FanIn, NOC + FanOut, NOC + NLOC, NOC + NOMI, NOC + NOPRM, NOC + NOPA, NOA + FanOut, NOA + NOAI, NOA + NOPRA, NOA + NOPA, NOA + NOPM, FanIn + FanOut, FanIn + NOMI, FanIn + NOPRA, FanIn + NOPRM, FanIn + NOPA, FanIn + NOPM, FanOut + NOAI, FanOut + NLOC, FanOut + NOM, FanOut + NOMI, FanOut + NOPRM, FanOut + NOPM, NOAI + NLOC, NOAI + NOMI, NOAI + NOPRM, NLOC + NOMI, NLOC + NOPRA, NLOC + NOPRM, NLOC + NOPA, NOM + NOPRM, NOM + NOPA, NOMI + NOPRA, NOMI + NOPRM, NOMI + NOPA, NOMI + NOPM	**83**

The influential metrics thus identified have increased information content as fault predictors, encompassing different aspects of the measurement of software characteristics. Brief description of metrics discussed in results is given in Table 1.

Referring to Table 7, while first considering CK metrics with interaction [CK (WI)]; coupling between objects (CBO), lack of cohesion of methods (LCOM), and response for class (RFC) metrics are influential in isolation, hence appearing individually and affirming the results reported by ( Gyimothy et al. 2005). In furtherance of this, other influential metrics derived in CK (WI) as shown in Table 7 are appearing as interacting terms.

The individual characteristics of LCOM measure the level of relatedness among the methods of a class, and those of CBO measure the dependence of this class to other classes. The interrelatedness of these individual metrics, i.e. CBO and LCOM, can be justified by the fact that they both share class attributes, member functions and the use of the attributes by these methods, consequently appearing as CBO + LCOM.

Weighted method per class (WMC) is the weighted sum of the complexity of the methods and both CBO and RFC are based on the invocation of a method from another class, thereby making them related to one another (RFC + WMC, CBO + RFC). Further, the interdependence of CBO and depth of inheritance tree (DIT) can be explained on account of the fact that the coupling between classes, arising from inheritance, will be higher for the classes which have high values of DIT ( Subramanyam and Krishnan 2003). CK metrics, in general, refer to the different aspects of a class design; that is identification, semantics and relationship with other classes and are often interrelated ( Chidamber et al. 1998).

Regarding the predictive capability of inheritance metrics i.e. DIT and number of children (NOC), contradictory results have been reported in literature ( Okutan and Yildiz 2012; Yu 2012; Basili et al. 1996; Gyimothy et al. 2005; Subramanyam and Krishnan 2003). Nevertheless, our results indicate that their combination (interaction) with other metrics like WMC, LCOM and RFC becomes a determining factor in the accuracy of the fault prediction model.

Other OO metrics used in this paper have the additional advantage of simplicity in the measurement of software characteristics; that is complexity, reusability, encapsulation and modularity. As is evident from Table 3, these metrics exhibit predictive power equivalent to CK metrics, if not better.

Similar to the argument presented for CK (WI), within OO (WI) in Table 7 dominant OO metrics in isolation are number of attributes (NOA), FanIn, FanOut, number of attributes inherited (NOAI), NLOC, number of methods (NOM), number of private methods (NOPRM) and number of public attributes (NOPA). Whereas FanOut, FanIn, NLOC, number of private attributes (NOPRA) and NOPRM metrics are more frequently used in interacting terms.

The majority (42 out of 83) of influential metrics considered under CK + OO (WI) is derived from the combination (with interaction) of CK and OO metrics. Subsequently, it has been observed that the metric FanOut appears in combination with all CK metrics, which further validates the applicability of inter-class metrics when used in combination. Out of 30 interacting OO metrics from within CK + OO (WI), metrics like NOPRA, NOPA, NOPRM, number of public methods (NOPM) and number of methods inherited (NOMI) frequently appear in combination. These primitive OO metrics quantify the basic building blocks of a typical object oriented software module and contribute significantly to the development of a fault prediction model.

The number of metrics within the superset of all interacting terms indicates a significant reduction in the total number of metrics to be considered in the design of a predictive model, which also maintains an adequate level of accuracy for all five software modules. Table 8 shows the statistics generated by only including those variables found in the superset for CK metrics, OO metrics and their combination. The value of statistical measure i.e. Adj. R^2^ is significantly consistent and acceptable (almost 90%) in comparison to the values obtained through total possible interacting terms for CK , OO and their combined metrics respectively. This elaborates and establishes the significance of the reduction in number of interacting metrics.

**Table 8:** **Statistical measures for superset of CK, OO metrics and their combination**

**CK metrics**				
Software modules	With superset of 11 interacting terms		With total 21 interacting terms	
Mylyn	0.1709	**0.1660**	0.1877	**0.1784**
PDE	0.0909	**0.0842**	0.0976	**0.0848**
Lucene	0.4010	**0.3913**	0.4202	**0.4020**
Equiox	0.6490	**0.6366**	0.6826	**0.6605**
Eclipse	0.5071	**0.5016**	0.5318	**0.5217**
**OO metrics**				
Software modules	With superset of 38 interacting terms		With total 66 interacting terms	
	R^2^	Adj R^2^	R^2^	Adj R^2^
Mylyn	0.3134	**0.2991**	0.3516	**0.3277**
PDE	0.6709	**0.6623**	0.7049	**0.6912**
Lucene	0.4918	**0.4622**	0.5774	**0.4996**
Equiox	0.7887	**0.7605**	0.8349	**0.7925**
Eclipse	0.5527	**0.5349**	0.6210	**0.5941**
**Combination of CK and OO metrics**				
Software modules	With superset of 83 interacting terms		With total 153 interacting terms	
	R^2^	Adj R^2^	R^2^	Adj R^2^
Mylyn	0.4156	**0.3883**	0.4784	**0.4316**
PDE	0.7204	**0.7040**	0.7683	**0.7419**
Lucene	0.5934	**0.5378**	0.6847	**0.5948**
Equiox	0.8580	**0.8089**	0.9384	**0.8830**
Eclipse	0.7217	**0.6964**	0.7933	**0.7558**

## Threats to validity

Certain issues that could have an effect on the results of the study and may have subsequently limited our interpretations were identified;

The scope of this paper is restricted to two-term interaction effects in the context of linear regression. Non-linear regression has other well developed heuristic based approaches of feature selection, which are beyond the scope of this paper.

In SWR a unique optimal subset of variables is presumed, however the presence of multiple optimal solutions cannot be denied. Thus, the process presented herein may be augmented by an additional step to identify the "best" of all the possible subsets, obtained after the slaying of a cycle of SWR.

Five different Java based software modules, each with a reasonable number of records, were considered in this study. In order to further support the derived results, software modules implemented in other programming languages may also be considered.

## Conclusion

The objective of this study was to find the set of influential interacting predictive variables in dealing with interaction based predictive modelling. A total of 17 metrics derived from the dataset taken were used in isolation, as well as in combination. Statistics generated reveal that the impact of interaction results in a fairly increased value of Adjusted R^2^, and this claim is further supported by calculations made for all five software modules in the given dataset. However, not all interactions are equally contributing. To find the most influential subset of interacting terms, SWR was conducted up to a 10% threshold of Adjusted R^2^ (up to 90% of its value) and the resulting [reduced] set of metrics which contribute the most towards prediction was thereby obtained. This reduced set of metrics derived, was further computed for all software modules in the dataset.

Adherence to the guidelines and methodology suggested in this article should assist readers in understanding interaction effects in fault prediction and finding an influential subset. A regression model containing interactive relationships has an edge over simple additive models, if not for the fact that it leads to the further requirement of deriving a reduced set of variables from the increased set obtained. The scope of this paper is, however, limited to interaction effects in the context of linear regression.

### Web sites

Bug Prediction Dataset: [online] http://bug.inf.usi.ch

## References

[CR1] Basili VR, Briand LC, Melo WL (1996). A validation of object-oriented design metrics as quality indicators. IEEE Trans Softw Eng.

[CR2] Bibi S, Tsoumakas G, Stamelos I, Vlahavas I (2008). Regression via Classification applied on software defect estimation. Expert Syst Appl.

[CR3] Briand LC, Wüst J, Daly JW, Victor Porter D (2000). Exploring the relationships between design measures and software quality in object-oriented systems. J Syst Softw.

[CR4] Chidamber SR, Kemerer CF (1994). A metrics suite for object oriented design. IEEE Trans Softw Eng.

[CR5] Chidamber SR, Darcy DP, Kemerer CF (1998). Managerial use of metrics for object-oriented software: An exploratory analysis. IEEE Trans Softw Eng.

[CR6] Cohen J, Cohen P, West SG, Aiken LS (2003). Applied multiple Regression/correlation analysis for the behavioral sciences.

[CR7] D’Ambros M, Lanza M, Robbes R (2010). An extensive comparison of bug prediction approaches. In Mining Software Repositories (MSR), 2010 7th IEEE Working Conference.

[CR8] D’Ambros M, Lanza M, Robbes R (2012). Evaluating defect prediction approaches: a benchmark and an extensive comparison. Empir Softw Eng.

[CR9] Denaro G, Pezzè M, Morasca S (2003). Towards industrially relevant fault-proneness models. Int J Softw Eng Knowl Eng.

[CR10] Draper N, Smith H (1981). Applied Regression Analysis.

[CR11] Goyal R, Chandra P, Singh Y (2013). “Impact of interaction in the combined metrics approach of fault prediction”. Softw Qual Prof.

[CR12] Gyimothy T, Ferenc R, Siket I (2005). Empirical validation of object-oriented metrics on open source software for fault prediction. IEEE Trans Softw Eng.

[CR13] Khoshgoftaar TM, Allen EB, Deng J (2002). Using regression trees to classify fault-prone software modules. IEEE Trans Reliab.

[CR14] Nagappan N, Ball T, Zeller A (2006). Mining metrics to predict component failures. In Proceedings of the 28th international conference on Software engineering.

[CR15] Okutan A, Yildiz OT (2012). Software defect prediction using Bayesian networks. Empirical Software Engineering.

[CR16] Pedhazur EJ (1997). Multiple regression in behavioral research: Explanation and prediction.

[CR17] Peters F, Menzies T, Marcus A (2013). Better cross company defect prediction. In Proceedings of the Tenth International Workshop on Mining Software Repositories.

[CR18] Radjenovic D, Herico M, Torkar R, Zivkovic A (2013). Software fault prediction metrics: A systematic literature review. Inf Softw Technol.

[CR19] Raj Kiran N, Ravi V (2008). Software reliability prediction by soft computing techniques. J Syst Softw.

[CR20] Runkler TA (2012). Data Analytics: Models and Algorithms for Intelligent Data Analysis.

[CR21] Subramanyam R, Krishnan MS (2003). Empirical analysis of ck metrics for object-oriented design complexity: Implications for software defects. IEEE Trans Softw Eng.

[CR22] Thwin MMT, Quah T-S (2005). Application of neural networks for software quality prediction using object-oriented metrics. J Syst Software.

[CR23] Yu L (2012). Using Negative Binomial Regression Analysis to Predict Software Faults: A Study of Apache Ant. Int J Inf Technol Comput Sci.

